# LncRNA SNHG17 promotes tumor progression and predicts poor survival in human renal cell carcinoma via sponging miR-328-3p

**DOI:** 10.18632/aging.203440

**Published:** 2021-09-08

**Authors:** Jie Wu, Gang Dong, Tingting Liu, Shaojin Zhang, Lulu Sun, Weijie Liang

**Affiliations:** 1 Department of Ultrasound Intervention, The First Affiliated Hospital, Zhengzhou University, Zhengzhou, He’nan Province, China; 2 Department of Urology Surgery, The First Affiliated Hospital, Zhengzhou University, Zhengzhou, He’nan Province, China; 3 Department of Gastrointestinal Surgery, The First Affiliated Hospital, Zhengzhou University, Zhengzhou, He’nan Province, China

**Keywords:** renal cell carcinoma, SNHG17, miR-328-3p, H2AX

## Abstract

Accumulating data shows that dysregulation of long non-coding RNAs (lncRNAs) are involved in human tumors' occurrence and progression. Small nucleolar RNA host genes (SNHGs) are recently revealed to play a carcinogenic role in various human neoplasms. However, the functions and underlying mechanisms of lncRNA SNHG17 in renal cell carcinoma (RCC) are still elusive. We analyzed the relationship between SNHG17 expression levels and clinicopathologic characteristics and prognosis in patients with RCC according to TCGA RNA-sequencing data and our cohort data. Loss-of-function and gain-of-function experiments were conducted to examine the biological behaviors of SNHG17 on RCC cell proliferation, migration, invasion, apoptosis, and tumor growth *in vivo*. The interaction between SNHG17, miR-328-3p, and Histone’sH2Avariant (H2AX) was verified by bioinformatics, dual-luciferase reporter gene, and RNA immunoprecipitation (RIP). Highly expressed SNHG17 was evident in RCC tissue samples and cell lines, and SNHG17 overexpression was related to advanced TNM stage and reduced relapse-free and overall survival of patients with RCC. Knockdown of SNHG17 prohibited malignant phenotypes, whereas ectopic SNHG17 expression showed the opposite effects. More importantly, SNHG17 could upregulate the expression of H2AX by acting as a miR-328-3p sponge. *In vivo* experiments confirmed that SNHG17 promoted the growth of RCC tumors. SNHG17/miR-328-3p/H2AXaxis might be involved in RCC progression, which provided a potential therapeutic target for RCC.

## INTRODUCTION

Clear cell renal cell carcinoma (ccRCC) represents the most common histological subtype of RCC, accounting for approximately 3% of all adult cancers [1]. RCC is characterized by a high rate of relapse and metastasis, which exerts a socio-economic burden on society and patients' families. Currently, approximately 30% of all RCC patients will display distant metastasis after tumor resection, with a 5-year survival rate of less than 20% [2, 3]. However, the pathogenesis and mechanisms of RCC progression remain elusive [4].

In the last few years, high-throughput transcriptome analysis showed that less than 10% of the total mammalian genome could be transcribed and encode proteins. More than 90% of the human genome is transcribed to non-coding RNA (ncRNA). Among these ncRNAs, long ncRNAs (lncRNAs) of >200 nt in length have been well studied in the development and invasion of human malignancies [5]. Accumulating and compelling evidence has indicated that lncRNAs modulate diverse biological processes, including transcriptional regulation, interaction with RNAs and protein complex at the posttranscriptional level, and epigenetic regulation [6, 7]. LncRNAs exert oncogenic or suppressive functions in various human cancers, including RCC [8–10]. LncRNA small nuclear RNA host gene 17 (SNHG17) is located on 20q11.23, with a 1,186 nt in length. Recently, aberrant expression of SNHG17 has been suggested to play vital roles in the development and progression of human carcinomas, such as gastric cancer, prostate cancer, non-small cell lung cancer (NSCLC), and breast cancer, etc [11–14]. However, to the best of our knowledge, the biological roles and underlying molecular mechanisms of SNHG17 in RCC remain unclarified.

In this study, we detected the expression levels and clinical significance of SNHG17 in human RCC tissue specimens. Then, we conducted loss-of- and gain-of-function analysis to explore the biological functions of SNHG17 inhuman RCC cell lines. Importantly, we placed a particular emphasis on the mechanism of SNHG17 in RCC.

## MATERIALS AND METHODS

### Cell culture

Four human RCC cell lines (786-O, ACHN, Caki-1, and 769-P) and human tubular epithelial cells (HK-2) were purchased from ATCC (Rockville, MD, USA). The four RCC cell lines were cultured in DMEM (Gibco, USA) supplemented with 10% fetal bovine serum (FBS) and 1% penicillin-streptomycin.HK-2 cells were cultured in keratinocyte serum-free medium (K-SFM) supplemented with 5% FBS.

### Plasmids construction

Small interfering RNAs (siRNAs) against SNHG17 (siSNHG17#1, siSNHG17#2), H2AX (siH2AX) and Myb (siMyb), as well as siRNA scramble control (siSCR), were generated from Genepharma (Shanghai, China). The cDNA of human SNHG17 was amplified and cloned into the vector pcDNA3.1 and termed pcDNA3.1/SNHG17. The sequence was confirmed by DNA sequencing. The microRNA inhibitor, mimics, and negative control (NC inhibitor and NC mimic) were generated from Ribobio (Guangzhou, China).

### Cell transfection

Transfection of plasmids was conducted using Lipofectamine 3000 Reagent (Invitrogen, Carlsbad, CA, USA). After transfection for 24 h, RCC cells were collected for subsequent analyses. The knockdown and overexpression efficiencies were evaluated by quantitative RT–PCR (qRT-PCR).

### Animal experiments

Short hairpin RNA (shRNA) targeting SNHG17 (shSNHG17) was produced by GenePharma (Shanghai, China). ACHN cells (5 × 10^6^) stably expressing shSNHG17, SNHG17, or control vectors, generated from Genepharma (Shanghai, China), were implanted subcutaneously into the dorsal flank regions of five-week old male nude mice (Shanghai lingchang Laboratory Animals, China; *n* *=* 5 in each group). All mice experiments had been approved by the Committee of the Ethics of Animal Experiments of Zhengzhou University. The mice's tumor growth was detected every five days and calculated according to the formula: width^2^ × length/2. One month after the inoculation, the mice were executed, and each tumor sample was harvested for further experiments.

### Patient samples

Eighty-four RCC tumor tissue (TT) samples and matched adjacent nontumor tissue (ANT) samples were collected from the Department of Urology, the First Affiliated Hospital of Zhengzhou University. Tissue samples were immediately snap-frozen in liquid nitrogen for long term preservation until RNA extraction. Based on the 2010 American Joint Committee on Cancer (AJCC) tumor-node-metastasis (TNM) classification, all patients were staged [15]. Detailed information was shown in Supplementary Table 1. The study was executed following the ethics committee of this hospital. All the patients provided written informed consent.

### Quantitative RT-PCR

Trizol reagent (Invitrogen) was used for RNA isolation. The Reverse Transcription Kit (Takara, Tokyo, Japan) was used for reverse transcription of extracted RNA. The expression of indicated genes was normalized to endogenous reference controls, GAPDH and U6, and was valued using 2^−ΔΔCt^ method [16]. Primer sequences were displayed in Supplementary Table 2. Primers for miR-328-3p and other candidate miRNAs were produced from RiboBio (Guangzhou).

### Western blot

RIPA lysis buffer (Thermo Scientific) containing 0.5% PMSF was used to separate the cells' proteins. Equivalent proteins (30 μg) were isolated on 10% SDS-PAGE gel and then transferred to PVDF membranes (Millipore, Billerica, MA, USA). After incubation in blocking solution for 1 h, proteins were immunoblotted with primary antibodies:H2AX (1:1000; Abcam, Cambridge, MA, USA) or β-actin (1:2000; Abcam, Cambridge, MA, USA) overnight at 4°C. Following incubation with the secondary antibody, the blots were finally detected using enhanced chemiluminescence (Pierce, Rockford, IL, USA). Protein expression levels were normalized to β-actin and quantitated with ImageJ (https://imagej.nih.gov/ij/).

### Subcellular fractionation and fluorescence *in situ* hybridization (FISH)

A PARIS Kit (Thermo Fisher Scientific, Waltham, Mass., USA) was used to isolate nuclear and cytoplasmic RNA, which were then detected by quantitative RT-PCR. GAPDH and U6 were served as endogenous reference controls in the cytoplasm and nucleus, respectively. The FISH assay was used to determine the subcellular location of SNHG17 in ACHN cells. The cell suspension was diluted to 100 cells/μL and seeded in the autoclaved glass slides. After cells were grown for 24 hours, cells were fixed in 4% paraformaldehyde at room temperature for 10 min. ACHN cells were then hybridized with fluorescein-labeled antisense RNA probes specific for SNHG17 (Gene Pharma) overnight at 37°C. Slides were rinsed, washed, and then counterstained with 4′-6′diamidino-2-phenylindole (DAPI). Images were acquired with a fluorescence microscope (IX70, Olympus, Japan).

### Cell proliferation

We used CCK8 (Dojindo, Japan) and Cell Titer-Glo luminescent cell viability kit (Promega Corporation, Madison, WI, USA) to examine cell proliferation. For CCK-8 assay, RCC cell lines (1.0 × 10^3^ cells per well) were cultured in a 96-well plate for 24 h after transfection. An enzyme-linked immunosorbent assay (ELISA) plate reader was then used to detect the absorbance of 575 nm at 0, 24, 48, and 72 h. For Cell Titer-Glo luminescent cell viability assay, the transfected RCC cells were seeded in 96-well plates at a density of 1 × 10^4^ cells/well in culture medium and cultured overnight at 37°C. Following this, 100 μl Cell Titer-Glo solution (Promega Corporation, Madison, WI, USA), mixed with the culture medium, was incubated for 20 min at room temperature, and the intensity of luminescence was detected.

### Caspases activity

RCC cell lines (786-O and ACHN), seeded in 6-well plates, were cultured at 37°C for 24 h. After the cells were collected and lysed in ice-cold cell lysis buffer (50 μl), caspase-3, -8, and -9 colorimetric assay kits (Abcam, Cambridge, MA, USA) were used to examine the activity of caspases. A microtiter plate reader (Benchmark, Bio-Rad, USA) at OD405 nm was used to read samples.

### Flow cytometry

Cell cycle distribution was detected using flow cytometry. After the RCC cells had been harvested, cells were fixed in cold ethanol at 4°C overnight. The fixed samples were incubated with RNase A (50 mg/mL, Beyotime) and propidium iodide (PI) (100 mg/mL, Beyotime) at 37°C for 30 min. FACSCalibur (BD Biosciences, USA) was used to collect cells, and cell cycle distribution was analyzed by FlowJo software 7.6.1 (Tree Star, USA).

### Scratch wound-healing assay

The scratch assay was conducted in 24-well plates with a serum-free medium until they reached 100% confluence, and then a wound on monolayer cells across each well's center was generated using a pipette tip. Then, cell motility was tested according to gap breadth at the indicated time points by a phase-contrast microscope (Nikon, Tochigi, Japan).

### Transwell assay

In a 24-well plate Trans well system (8-μm pore size, BD Biosciences, USA), RCC cell lines (4 × 10^4^) in serum-free medium were loaded to the upper chamber (500 μL for each chamber) with Matrigel-uncoated (for migration) or -coated membrane (for invasion). Culture medium supplemented with 20% FBS was loaded to the lower chamber. Following incubation for 24 hours, cells on the lower surface were immobilized in 4% paraformaldehyde solution and stained with 0.1% crystal violet solution. A light microscope (Olympus, Tokyo, Japan) was employed to count the cells and take pictures with three independent fields.

### Bioinformatics analysis, luciferase assay and RNA immunoprecipitation (RIP)

Data mining and bioinformatics analyses were performed following Star Base v3.0 (https://web.archive.org/web/20110222111721/http://starbase.sysu.edu.cn/), the Lnclocator [17], LNCipedia (https://lncipedia.org), miRDB (http://www.mircode.org), and UALCAN (http://ualcan.path.uab.edu/index.html). The predicted binding sites of miR-328-3p with SNHG17 were obtained from online software StarBase v3.0. Mutant-type (mut) vectors (SNHG17 mut and H2AX mut) were produced by a Mutagenesis Kit (QIAGEN, California, USA). These vectors were cloned into pGL3-Basic luciferase plasmids (Promega, Madison, WI, USA), and were then co-transfected into RCC cells using Lipofectamine 3000 Reagent (Invitrogen, Carlsbad, CA, USA). Following transfection for 36 hours, luciferase activities were measured in RCC cells using a luciferase assay system (Promega).

To perform RIP assay, we utilized an EZ-Magna RIP™ RNA-Binding Protein Immunoprecipitation Kit (Millipore). After RCC cell lines (786-O and ACHN) were lysed in RIP buffer, precipitated RNAs were acquired from the cell lysate supernatant using magnetic beads preincubated with antibodies (Ago2, or control IgG). The purified RNAs were analyzed by qRT-PCR.

### Biotinylated RNA pull-down assay

RCC cells (786-O and ACHN, 1 × 10^6^) were transfected with biotinylatedSNHG17 or control (50 nmol/L) using Lipofectamine 3000. Following culture for 48h, cells were pelleted at 1000 rpm, and then cell pellets were resuspended in 0.8 ml lysis buffer. Cell lysates were acquired by centrifugation at 10,000 g. Simultaneously, streptavidin- coupled magnetic beads (Invitrogen) were coated with yeast tRNA (Invitrogen) and incubated at 4°C for 2.5 hours. Then the beads were washed with splitting buffer and resuspended with lysis buffer. Sample lysates were mixed with pre-coated beads and incubated overnight at 4°C. The binding RNAs were washed and eluted, and then the expression levels of RNAs were analyzed using qRT-PCR assay.

### Statistical analysis

Experiments were executed in triplicate, and the data were shown as mean ± SD. Statistical analyses and graphs were performed using Statistical Program for Social Sciences (SPSS) 19.0 (SPSS Inc., Chicago, IL, USA) and GraphPad Prism version 8.0 (GraphPad Inc., La Jolla, CA, USA). When appropriate, the chi-squared test, student’s *t* test, variance analysis, and Spearman correlation test were used. Survival analyses, including overall survival (OS) and relapse-free survival (RFS), were conducted using the Kaplan–Meier curves and cox’s proportional hazards regression model. *P* < 0.05 was considered statistically significant.

### Availability of data and materials

The data sets used and/or analyzed during the current study area available from the corresponding author on reasonable request.

### Ethics approval and consent to participate

The Ethics Committee approved the present study of the First Affiliated Hospital of Zhengzhou University. The research has been carried out following the World Medical Association Declaration of Helsinki.

## RESULTS

### Expression of SNHG17 is enhanced in RCC tissues and associated with poor prognosis

**Figure 1:**
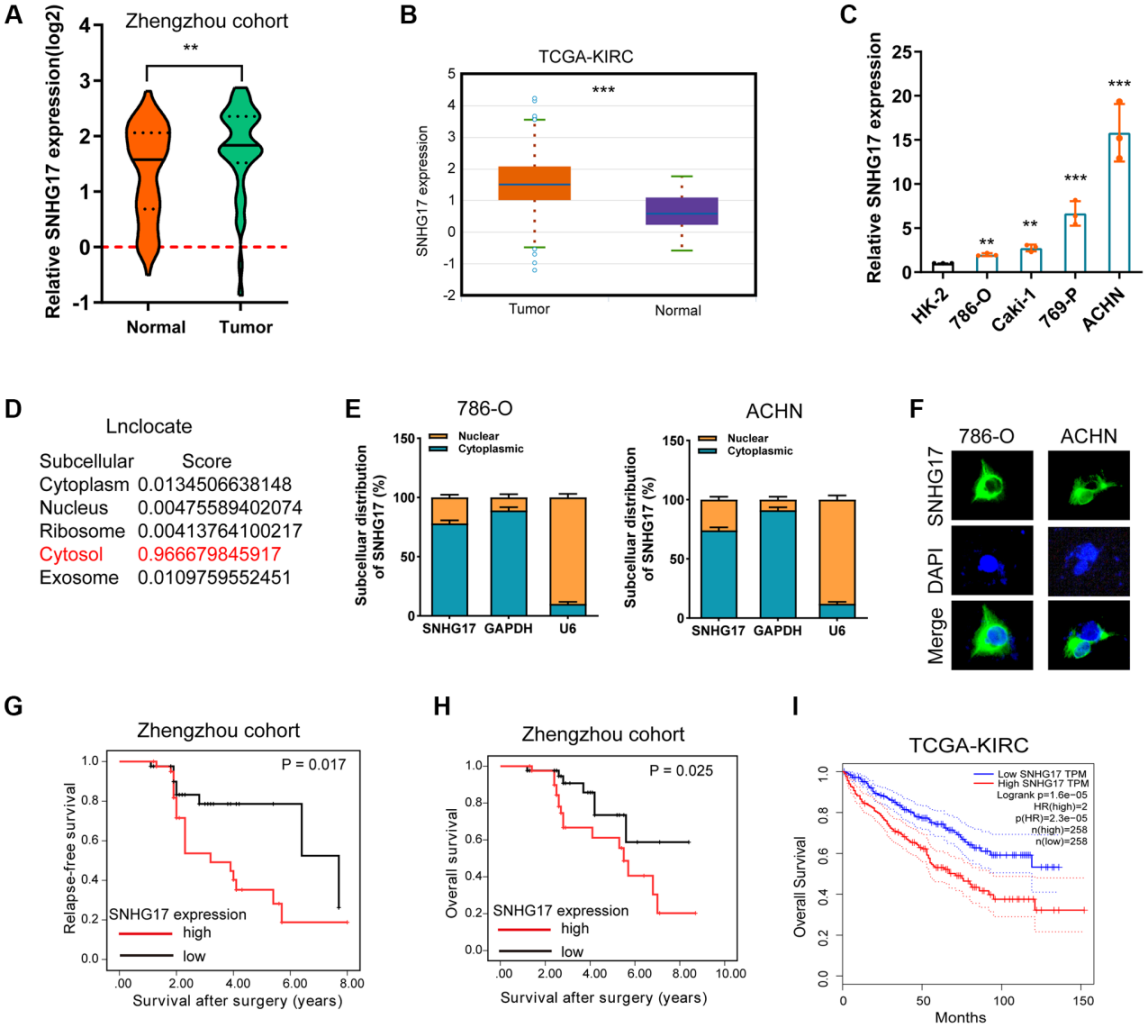
**Expression of SNHG17 is enhanced in RCC and associated with poor prognosis.** (**A–B**) qRT-PCR assay analysis of SNHG17 expression in human RCC tissues in patients from the Zhengzhou cohort (**A**) and TCGA-KIRC dataset (**B**). (**C**) qRT-PCR assay analysis of SNHG17 expression in RCC cell lines. (**D**) Predicted subcellular localization of SNHG17 using the "lnclocator" algorithm. (**E**) qRT-PCR assay analysis of the subcellular localization of SNHG17 in 786-O and ACHN cells. GAPDH and U6 served as a cytoplasmic and nuclear localization marker, respectively. (**F**) FISH assay analysis of the subcellular localization of SNHG17 in ACHN cells. (**G**–**H**) Kaplan-Meier survival curves showed that high expression of SNHG17 was related to dismal relapse-free (**G**) and overall survival (**H**) in patients with RCC from the Zhengzhou cohort. (**I**) High expression of SNHG17 was significantly associated with poor overall survival according to TCGA-KIRC dataset. RCC, renal cell carcinoma; TCGA, the cancer genome atlas; KIRC, kidney renal clear cell carcinoma; TT, tumor tissue; ANT, adjacent nontumor tissue. ^*^P < 0.05; ^**^P < 0.01; ^***^P < 0.001.

To test the expression status of SNHG17 in RCC, we conducted qRT-PCR assay to compare SNHG17 expression levels between RCC tumor tissues (*n* = 84) and corresponding ANT. Results showed that SNHG17 expression level was significantly higher in tumor tissues than their ANTs (*P* < 0.001, Figure 1A). We also explored SNHG17 level in the cancer genomic atlas (TCGA)-kidney renal clear cell carcinoma (KIRC) datasheet, and the results were consistent with our findings (Figure 1B). Besides, the expression levels of SNHG17 were markedly increased in four RCC cell lines compared with the HK-2 cell line (Figure 1C). To explore the subcellular location of SNHG17, we used the “lnclocator” online tool, [17] the cellular fractionation experiments, and LncRNA FISH assay, which showed that SNHG17 was preferentially localized to the cytoplasm of 786-O and ACHN cell lines (Figure 1D–1F). Meanwhile, the protein-coding capacity of SNHG17 was evaluated by LNCipedia (https://lncipedia.org), which validated SNHG17 as a non-coding RNA (Supplementary Figure 1A). Also, the secondary structure of SNHG17 is predicted by RNAfold Webserver (http://rna.tbi.univie.ac.at/) (Supplementary Figure 1B).

The correlation between SNHG17 expression and clinicopathologic features in 84 RCC samples was then analyzed. By dividing into two groups according to the median value of SNHG17 level, we found that high expression of SNHG17 was significantly associated with big tumor size, lymph node invasion, distant metastasis, and relapse status, but was not associated with poor tumor grade (Supplementary Figure 2A–2E). Kaplan-Meier analysis found that high expression of SNHG17 was indicated dismal RFS (Figure 1G; *P* = 0.017) and OS (Figure 1H; *P* = 0.025) in the Zhengzhou cohort. Results from TCGA-KIRC dataset also confirmed our findings (Supplementary Figure 2F and Figure 1I). The further cox regression analyses suggested that high expression of SNHG17 was an independent indicator for dismal prognosis (for RFS, hazard ratio [HR] = 1.89, 95% confidence interval [CI]: 1.04–3.44; for OS, HR = 1.72, 95% CI, 1.03–2.87; Table 1). These data demonstrated that SNHG17 might function as an oncogenic lncRNA in RCC.

**Table 1:** Univariate and multivariate analyses of prognostic factors in patients with renal cell carcinoma.

**Parameters**	**Univariate analysis hazard ratio (95% CI)**	***P* value**	Multivariate analysis, hazard ratio (95% CI)^*^	***P* value**
**Relapse-free survival**				
**T stage**				
T3 + T4/T1 + T2	2.13 (1.01–4.48)	0.046	1.83 (1.00–3.35)	0.050
**N stage**				
N1/N0	1.85 (1.12–3.05)	0.016	1.63 (1.05–2.52)	0.028
**M stage**				
M1/M0	1.69 (1.15–2.47)	0.007	1.48 (1.03–2.12)	0.032
**Grade**				
3 + 4/1 + 2	1.43 (1.01–2.04)	0.047	1.65 (0.88–3.09)	0.117
**SNHG17**				
High/low	2.49 (1.13–5.50)	0.024	1.89 (1.04–3.44)	0.037
**Overall survival**				
**T stage**				
T3 + T4/T1 + T2	1.88 (1.05–3.26)	0.003	1.68 (1.08–2.61)	0.021
**N stage**				
N1/N0	2.58 (1.11–6.01)	0.028	1.71 (1.03–2.85)	0.039
**M stage**				
M1/M0	1.54 (0.75–3.15)	0.238		
**Grade**				
3 + 4/1 + 2	1.78 (1.05–3.01)	0.031	1.61 (1.15–2.25)	0.005
**SNHG17 expression**				
High/low	2.63 (1.09–6.35)	0.032	1.72 (1.03–2.87)	0.038

NOTE: Each patient was staged according to 2010 AJCC TNM classification.

### Upregulation of SNHG17 drives malignant phenotype of RCC

**Figure 2:**
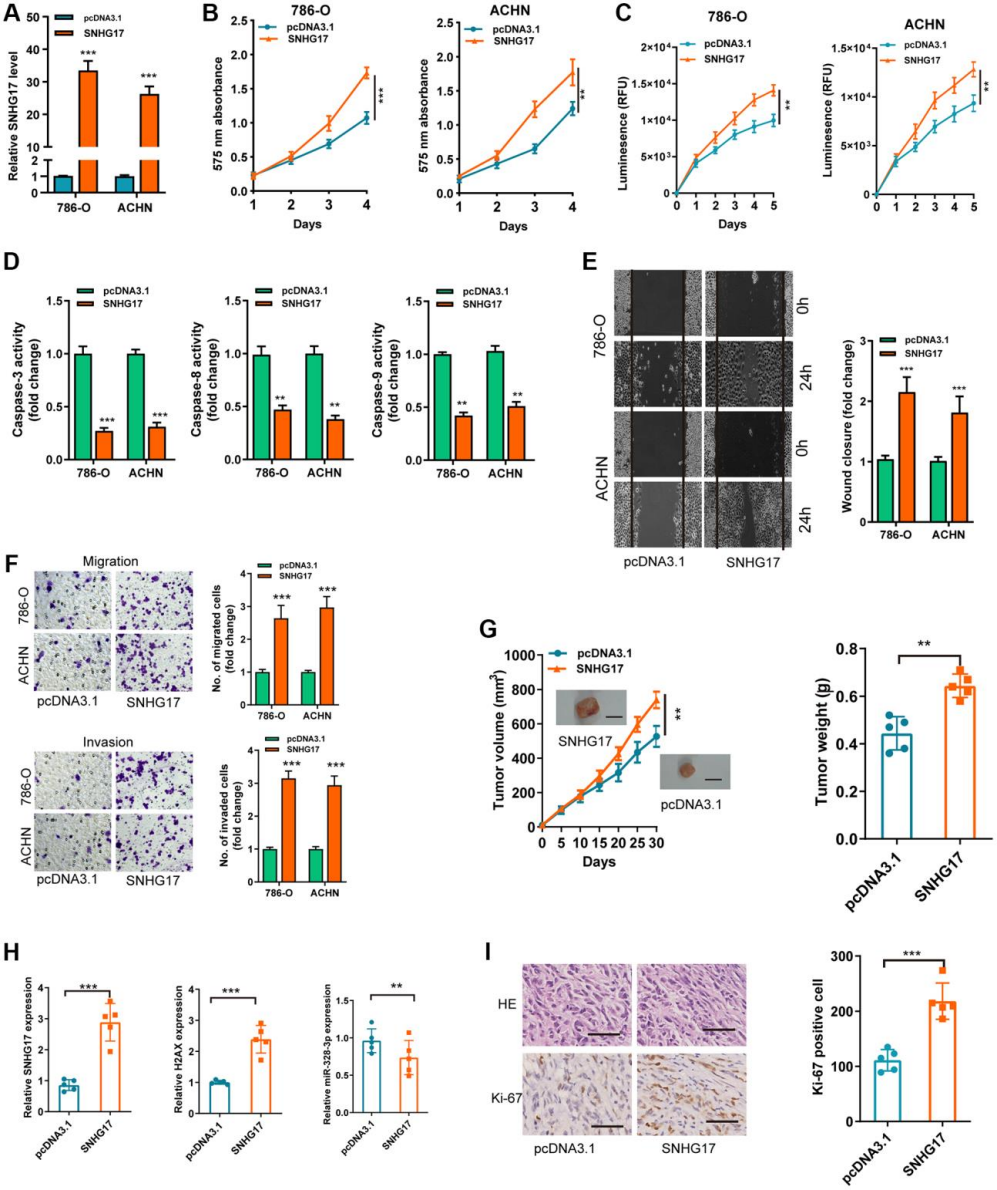
**Upregulation of SNHG17 drives malignant phenotype of RCC.** (**A**) qRT-PCR assay analysis of the expression of SNHG17 in 786-O and ACHN cell lines transfection with pcDNA3.1/SNHG17 or pcDNA3.1. (**B**–**C**) CCK-8 assay (**B**) and Cell Titer-Glo Luminescent cell viability assay (**C**) analysis of the proliferative ability of 786-O and ACHN cells transfected with the indicated vectors. (**D**) The activity of caspase-3, -8, and -9 assay analysis of cell apoptosis of786-O and ACHN cells transfected with the indicated vectors. (**E**) The wound healing assay analysis of cell migration in 786-O and ACHN cell lines transfected with the indicated vectors. (**F**) The transwell assay analysis of cell migration and invasion in 786-O and ACHN cell lines transfected with the indicated vectors. (**G**) SNHG17 overexpression remarkably increased the volume and weight of tumor xenograft. Scale bar, 1.0 cm. (**H**) qRT-PCR assay analysis of expression of SNHG17, H2AX and miR-328-3p in the tumor xenograft. (**I**) Immunohistochemistry staining of Ki-67 in the tumor xenograft. Scale bar, 200 μm. Data are presented as means ± standard deviation from triplicate experiments. A *t*-test was used to evaluate the statistical significance as compared to the control. RCC, renal cell carcinoma. ^*^P < 0.05; ^**^P < 0.01; ^***^P < 0.001.

To detect the biological functions of SNHG17 in RCC cell lines, we transfected pcDNA3.1/SNHG17 and control (pcDNA3.1) into 786-O and ACHN cell lines. After transfection for 48 hours, qRT-PCR analysis confirmed that upregulated SNHG17 levels were clarified in 786-O and ACHN cell lines (Figure 2A). CCK-8 and Cell Titer-Glo Luminescent cell viability assays found that SNHG17 overexpression significantly drove cell viability in 786-O and ACHN cell lines compared with the pcDNA3.1 group (Figure 2B–2C). Further, overexpression of SNHG17 significantly limited RCC cell apoptosis, as determined by activities of caspase-3, -8, and -9 assays (Figure 2D). To ascertain the role of SNHG17in cell cycle of RCC cells, flow cytometry was performed to determine the percentage of cells in phase S, with results indicating that SNHG17 overexpression drastically enhanced the cell percentage in phase S (Supplementary Figure 3). Also, stretch wound healing and transwell experiments validated the markedly increased migratory and invasive ability due to SNHG17 overexpression (Figure 2E–2F). To validate the functions of SNHG17 in the growth of RCC *in vivo, w*e performed a mouse xenograft experiment. Compared with the negative control groups, the volume and weight of tumor xenograft were remarkably increased in the SNHG17 overexpression groups (Figure 2G). Additionally, qRT-PCR assays verified the increased expression of SNHG17 and H2AX and decreased expression of miR-328-3p in the overexpression groups (Figure 2H). IHC staining of Ki-67 clarified that SNHG17 overexpression increased tumor proliferation (Figure 2I). These results exhibited that upregulation of SNHG17 could promote RCC cells’ malignant phenotype.

### SNHG17 depletion suppressed malignant phenotype of RCC

**Figure 3:**
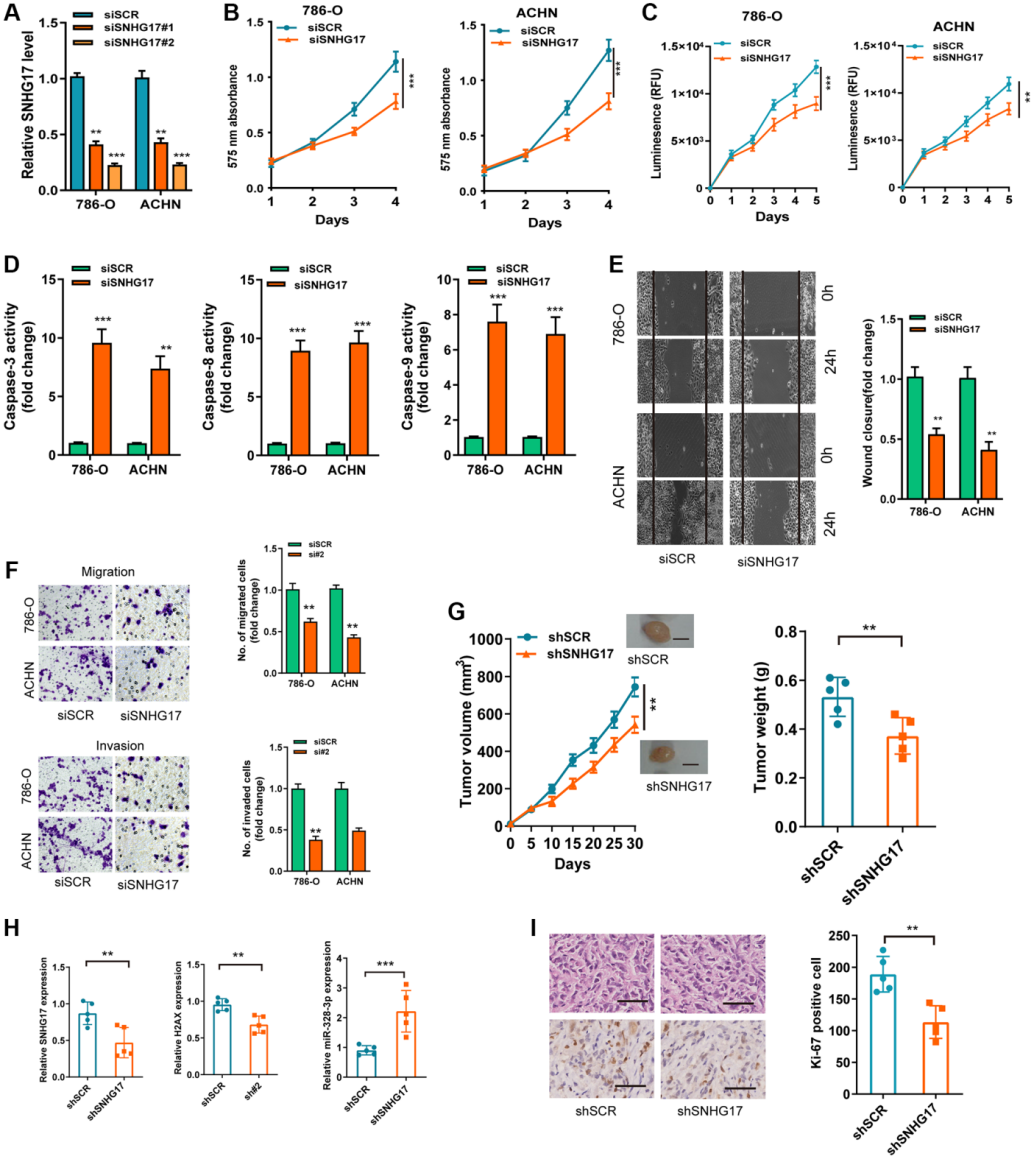
**SNHG17 depletion suppressed malignant phenotype of RCC.** (**A**) qRT-PCR assay analysis of the levels of SNHG17 in 786-O and ACHN cell lines transfected with two parallel siSNHG17 (#1 and #2) or siSCR. (**B**–**C**) CCK-8 assay (**B**) and CellTiter-Glo Luminescent cell viability assay (**C**) analysis of the proliferative ability of RCC cells transfected with the indicated vectors. (**D**) The activity of caspase-3, -8, and -9 assay analysis of cell apoptosis of RCC cells transfected with the indicated vectors. (**E**) The wound healing assay analysis of cell migration of RCC cells transfected with the indicated vectors. (**F**) The transwell assay analysis of cell migration and invasion in RCC cell lines transfected with the indicated vectors. (**G**) SNHG17 overexpression remarkably increased the volume and weight of tumor xenograft. Scale bar, 1.0 cm. (**H**) qRT-PCR assay analysis of expression of SNHG17, H2AX and miR-328-3p in the tumor xenograft. (**I**) Immunohistochemistry staining of Ki-67 in the tumor xenograft. Scale bar, 200 μm. Data are presented as means ± standard deviation from triplicate experiments. A *t*-test was used to evaluate the statistical significance as compared to the control. SCR, scramble control; RCC, renal cell carcinoma. ^**^P < 0.01; ^***^P < 0.001.

We then used two siRNAs targeting the SNHG17 (siSNHG17#1 and siSNHG17#2) for the silencing experiments, and qRT-PCR assay confirmed the higher knockdown efficiency of siSNHG17#2than siSNHG17#1 (Figure 3A). So, we selected siSNHG17#2 to conduct the following silencing experiments. The CCK-8 and Cell Titer-Glo luminescent cell viability assays showed an evident decrease in RCC cells' viability (Figure 3B–3C). Moreover, the silencing of SNHG17 resulted in enhanced activity of caspase-3, -8, and -9 and reduced the cell percentage in phase S of 786-O and ACHN cell lines (Figure 3D and Supplementary Figure 4). Furthermore, the downregulation of SNHG17 remarkably suppressed the migration and invasion of 786-O and ACHN cell lines (Figure 3E–3F). The further mouse xenograft experiment also validated that the downregulation of SNHG17 inhibited RCC cells' malignant phenotype *in vivo* (Figure 3G–3I).

### MiR-328-3p is sponged by SNHG17

**Figure 4:**
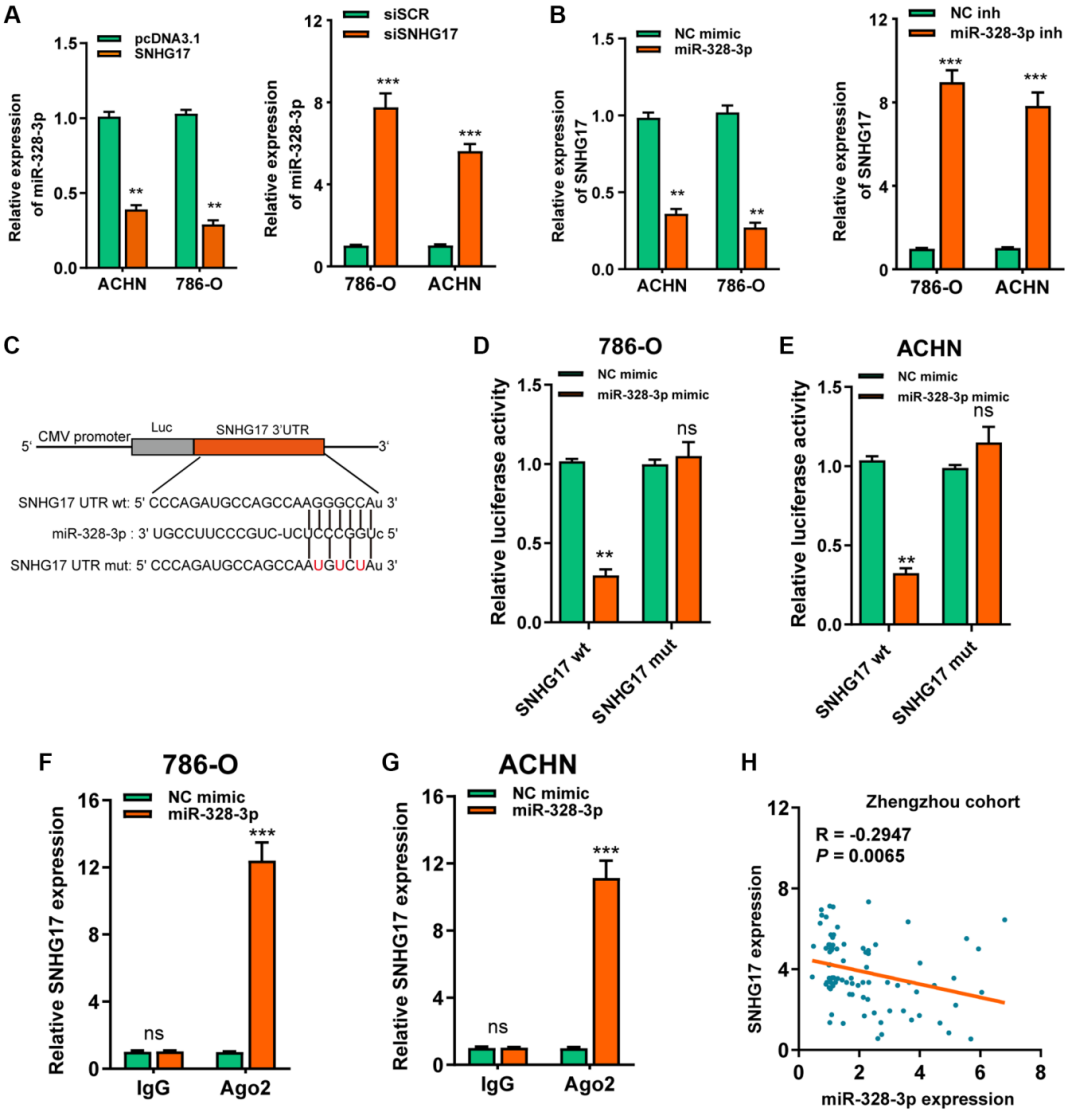
**MiR-328-3p is sponged by SNHG17.** (**A**–**B**) qRT-PCR assay analysis of the expression of miR-328-3p (**A**) and SNHG17 (**B**) in 786-O and ACHN cell lines after transfection with the indicated vectors. (**C**) The potential binding sites of SNHG17 and miR-328-3p were predicted by StarBase v3.0. (**D**–**E**) Luciferase activity was analyzed in 786-O and ACHN cells co-transfected with miR-328-3p mimic or NC mimic and SNHG17 wt or mut luciferase reporter vector. (**F**–**G**) The level of SNHG17 enriched by Ago2 antibody was detected in 786-O and ACHN cells transfected with miR-328-3p mimic or NC mimic. (**H**) Spearman’s correlation analysis of the correlation between SNHG17 and miR-328-3p in RCC tissues from the Zhengzhou cohort. Data are presented as means ± standard deviation from triplicate experiments. A *t*-test was used to evaluate the statistical significance as compared to the control. NC, negative control; SCR, scramble; RCC, renal cell carcinoma; ns, not significant. ^*^P < 0.05; ^**^P < 0.01; ^***^P < 0.001.

As denoted above, SNHG17 was mainly localized in the cytoplasm, we therefore want to know whether SNHG17 acted as a miRNA sponge. After bioinformatics analysis according to the Star Base v3.0, miR-328-3p, miR-23b-3p, miR-451a were suggested as potential miRNAs that might bind SNHG17. Furthermore, the three microRNAs expression possessed a negative correlation with SNHG17 in TCGA-KIRC dataset (Supplementary Figure 5A). Besides, the RNA pull-down assay showed that only miR-328-3p was markedly pulled down by biotinylated SNHG17 (Supplementary Figure 5B). In addition, we revealed that the up-regulation of SNHG17 inhibited miR-328-3p expression, while downregulation of SNHG17 increased the expression of miR-328-3p in 786-O and ACHN cells (Figure 4A). Nevertheless, no changes were found in the expression of miR-23b-3p or miR-451a when manipulating the expression of SNHG17 in RCC cells (Supplementary Figure 5C). Furthermore, miR-328-3p mimic led to reduced expression of SNHG17, and miR-328-3p inhibitor increased the expression of SNHG17 (Figure 4B). So, we selected miR-328-3p for further investigation. We conducted luciferase and RIP assays to validate this binding in RCC cells. As depicted in Figure 4C–4E, the upregulation of miR-328-3p resulted in decreased luciferase activity in SNHG17 wt group, while it did not affect luciferase activity in SNHG17-mut group. Furthermore, transfection of miR-328-3p mimic in RCC cells enhanced SNHG17 enrichment by the Ago2 antibody, whereas the IgG group showed little enrichment of SNHG17. Figure 4F–4G Finally, Spearman's correlation analysis suggested a negative association between miR-328-3p and SNHG17 expression in RCC tissues from the Zhengzhou cohort (*R* = −0.2947, *P* = 0.0065; Figure 4H).

### SNHG17 enhanced the expression of H2AXvia sponging miR-328-3p

**Figure 5:**
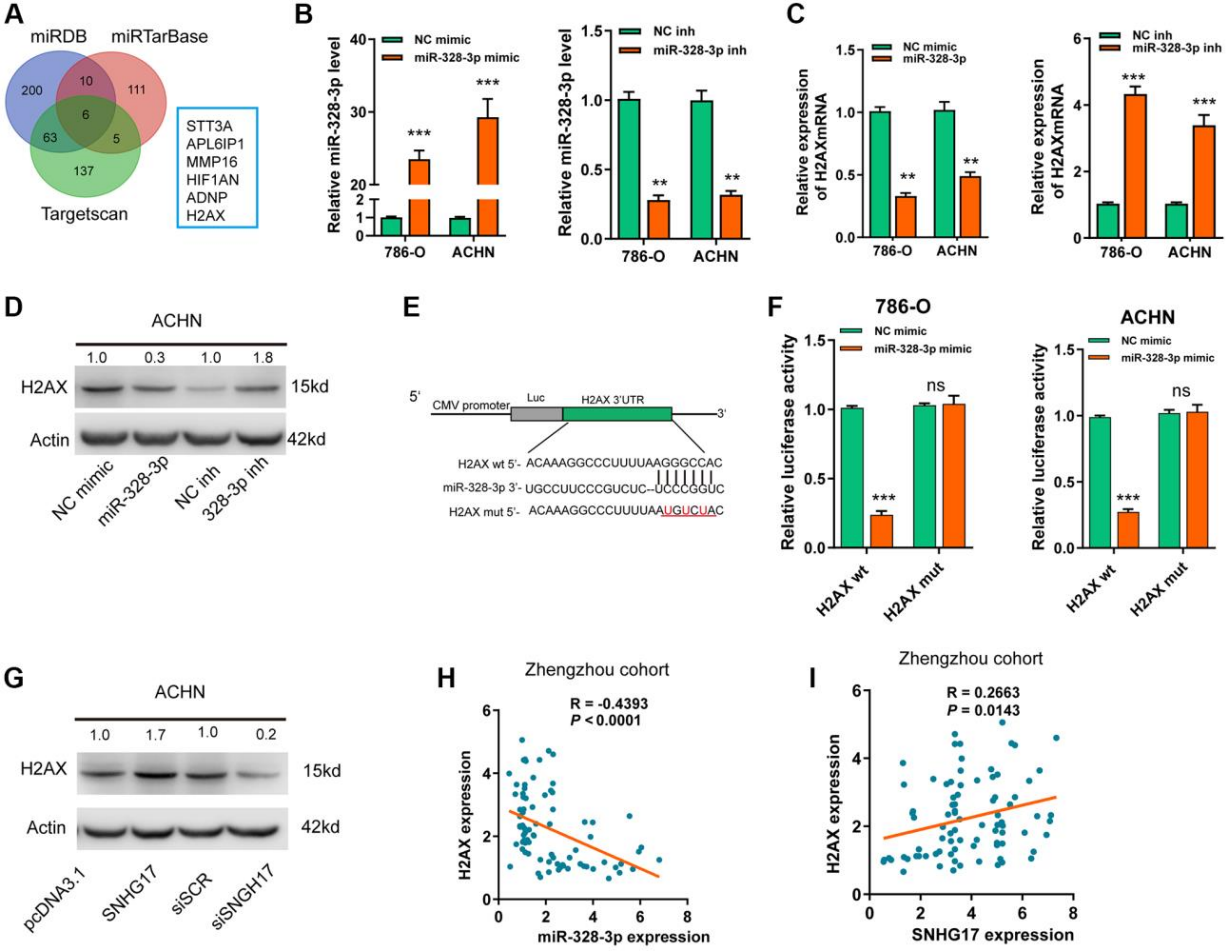
**SNHG17 enhanced the expression of H2AX via sponging miR-328-3p.** (**A**) A Venn diagram showed the number of genes identified as potential targets of miR-328-3p according to the three online tools. (**B**) qRT-PCR assay analysis of miR-328-3p expression in RCC cell lines after transfection of the indicated vectors. (**C**–**D**) qRT-PCR and western blot assay analysis of the expression levels of H2AX mRNA (**C**) and protein (**D**) in 786-O and ACHN cell lines after transfection with the indicated vectors. (**E**) The miR-328-3p putative binding sequences and corresponding mutant sites of H2AX 3′UTR. (**F**) The luciferase activity in RCC cell lines after co-transfection with miR-328-3p mimic or NC mimic and wt or mut constructs of H2AX 3′UTR. (**G**) western blot assay analyses of the expression levels of H2AX protein in RCC cell lines after transfection with the indicated vectors. (**H**) Spearman’s correlation analysis of the correlations between expression levels of miR-328-3p (**H**) or SNHG17 (**I**) and H2AX in RCC tissues from the Zhengzhou cohort. Data are presented as means ± standard deviation from triplicate experiments. Wt, wild-type; mut, mutant-type; SCR, scramble control; RCC, renal cell carcinoma; ns, not significant. ^*^P < 0.05; ^**^P < 0.01; ^***^P < 0.001.

Based on 3 online tools (targetscan7.1, miRDB, miRTarbase), we identified6 common targets that shared the regulation of miR-328-3p with SNHG17 (Figure 5A). Among the 6 genes, H2AX was upregulated and associated with the advanced TNM stage according to the dataset from TCGA-KIRC (Supplementary Figure 6A–6B). In addition, high expression of H2AX indicated poor prognosis (Supplementary Figure 6C). After successful transfection of miR-328-3p mimic or inhibitor in RCC cell lines (Figure 5B), we found that ectopic expression of miR-328-3p inhibited, while knockdown of miR-328-3p markedly increased, the mRNA (Figure 5C) and protein (Figure 5D) expression levels of H2AX. In Figure 5E, the binding site between H2AX and miR-328-3p was exhibited. Co-transfection of miR-328-3p mimic and H2AX wt 3′-UTR resulted in the depressed luciferase activity, but no evident changes were shown in cells co-transfected with miR-328-3p mimic andH2AX mut 3′-UTR (Figure 5F). Furthermore, SNHG17 upregulation promoted HAX expression levels at both mRNA and protein levels, while SNHG17 downregulation led to decreased HAX expression (Supplementary Figure 6D–6E and Figure 5G). Recently, a phosphorylated form of H2AX, named gamma-H2AX, has become a powerful tool to monitor DNA double stranded breaks (DSBs) [18]. Therefore, we examined whether SNHG17 expression affected gamma-H2AX expression levels. The data indicated that overexpression of SNHG17 facilitated, while knockdown of SNHG17 suppressed, gamma-H2AX expression in ACHN cells, suggesting an effect of SNHG17 on DSBs in RCC cells (Figure 5G). Also, Spearman’s correlation analysis showed that H2AX expression had a negative relation with miR-328-3p but a positive relation with SNHG17 expression in RCC tissues from the Zhengzhou cohort (Figure 5H–5I) and TCGA-KIRC dataset (Supplementary Figure 6F–6G). Together, these results clarified a regulatory axis ofSNHG17/miR-328-3p/H2AXin RCC cells.

### MiR-328-3p reverses SNHG17-induced tumor-promoting effects of RCC

**Figure 6:**
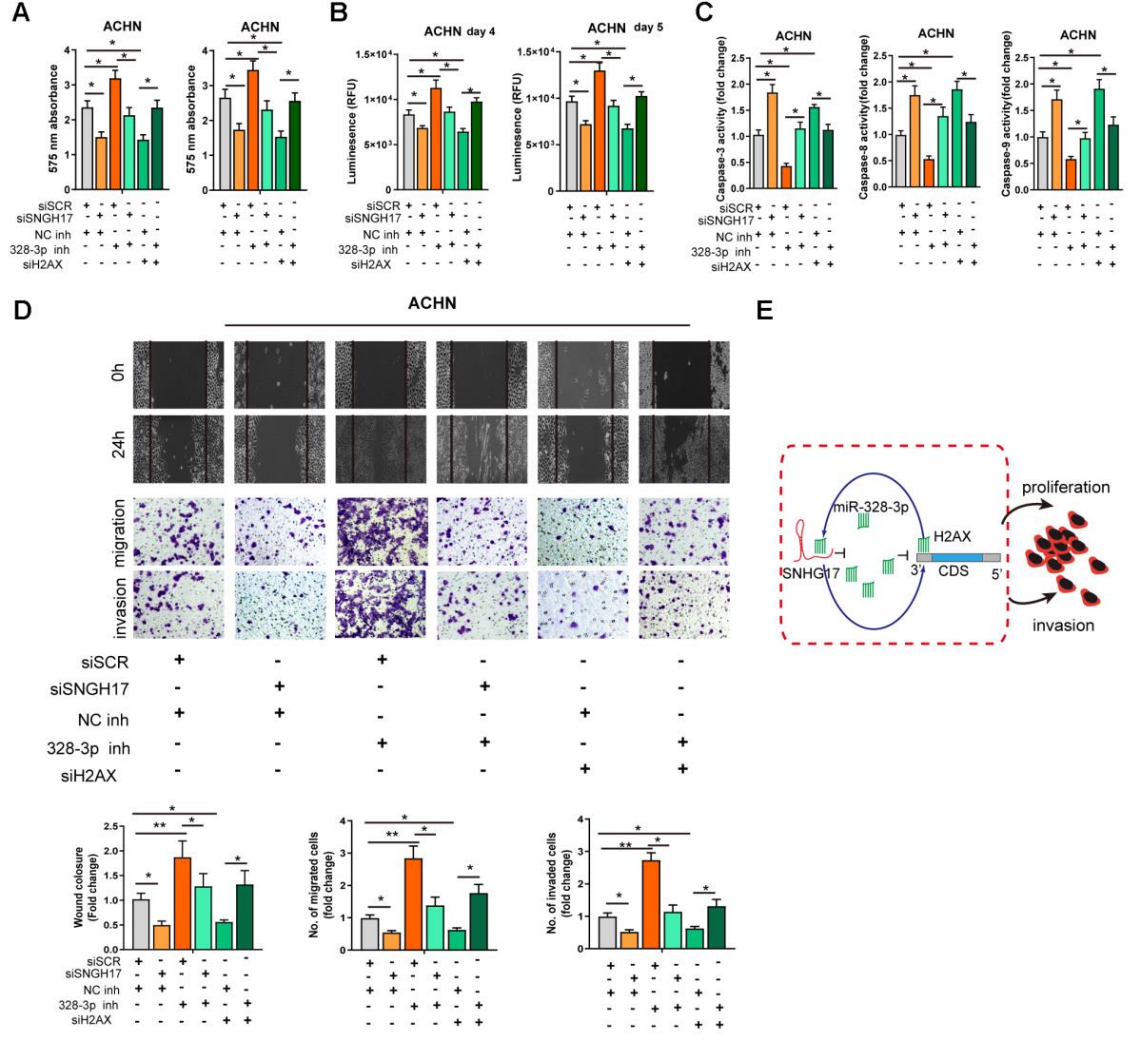
**MiR-328-3p reverses SNHG17-induced tumor-promoting effects of RCC.** (**A**–**D**) CCK-8 assay, CellTiter-Glo Luminescent cell viability assay, activity of caspase-3, 8, and 9 assay, wound healing and transwell assay were performed in ACHN cells transfected with siRNA scramble control (siSCR), siRNA against SNHG17 (siSNHG17) or H2AX (siH2AX), and miR-328-3p inhibitor or negative control inhibitor (NC inh) by special commercial kits. (**E**) Proposed model of the mechanism underlying the expression and function of SNHG17 in RCC cell. RCC, renal cell carcinoma. ^*^P < 0.05; ^**^P < 0.01; ^***^P < 0.001.

We performed rescue experiments to explore the functional relationship ofSNHG17/miR-328-3p/H2AXaxis in RCC cells. As exhibited in Figure 6A–6D, depletion of SNHG17 or H2AX significantly suppressed the cell viability, migration, and invasion but facilitated apoptosis of ACHN cells. Downregulation of miR-328-3p alone remarkably elevated the cell viability, migration, and invasion but inhibited apoptosis of ACHN cells. After ACHN cells were co-transfected with miR-328-3p inhibitor and siRNA targeting SNHG17 or H2AX, we found that depletion of miR-328-3p counteracted the inhibitory effects induced by SNHG17 or H2AXsilencing. In summary, our investigation confirmed that SNHG17 could enhance H2AX expression via sponging miR-328-3p and subsequently promotes the progression of RCC (Figure 6E).

## DISCUSSION

In recent decades, increasing evidence has uncovered that lncRNAs can exert crucial effects on tumor progression, metastasis, and drug resistance [2, 8, 19]. However, only a small proportion of lncRNAs and their functions in the tumor have been well clarified. Herein, our team examined the clinical significance and biological roles of lncRNA SNHG17 in RCC patients and RCC cell lines. SNHG17 was demonstrated to promote RCC cells’ malignant phenotypes. According to the TCGA dataset and our cohort, high expression of SNHG17 was closely correlated to poor survival and tumor recurrence. Mechanically, our data validated that SNHG17 can sponge miR-328-3p through acting as a ceRNA and then elevate H2AX expression levels, drive RCC tumor progression.

LncRNA SNHG17 is recently indicated to play a critical role in the development and progression of several types of human malignancies [11–14]. For example, Xu et al. showed that gene amplification drove SNHG17 promoted proliferation and invasion and inhibited apoptosis of NSCLC cell lines [14]. In gastric cancer, Han and his coauthors found that lncRNA SNHG17 was upregulated by Helicobacter pylori infection, a well-known risk factor for gastric cancer, and high expression of SNHG17 was related todismalOS in patients with stomach cancer. In addition, overexpression of SNHG17 markedly altered the DNA repair system, which is essential for the maintenance of genomic stability [20]. According to the public database and our data, the present investigation revealed that expression levels of SNHG17 were markedly enhanced in RCC tissues and RCC cell lines. Specifically, high SNHG17 expression levels were closely associated with advanced pathological features (tumor size, lymph node invasion, and distant organ metastasis) and the dismal survival of RCC patients. The further gain-of-function and loss-of-function experiments determined that SNHG17 enhanced malignant behaviors in RCC cells. The mouse xenograft model *in vivo* suggested that the depletion of SNHG17 prohibited tumorigenesis, whereas overexpression of SNHG17 promoted tumor growth. These experiments revealed that SNHG17 acted as an oncogene in RCC and promoted tumor progression.

In recent years, accumulating evidence has implicated that lncRNAs exert biological roles by functioning as ceRNAs for sponging miRNA to regulating their target genes in human cancers [21, 22]. For instance, SNHG17, as a sponge of miR-506-3p, was clarified to upregulate the expression of CTNNB1 in glioma [23]. Furthermore, miR-124-3p is proven to be a sponge of SNHG17 in breast cancer cell lines [11]. In the present experiment, we worked hard to examine whether SNHG17 may also function as a ceRNA to regulate RCC’s tumorigenesis and progression. Using the bioinformatics database, the separation of nuclear and cytoplasmic RNA assays, and FISH experiments, we found that SNHG17 was mainly located in the cytoplasm of RCC cell lines and contained theoretical binding sites of miR-328-3p in the 3′ region of SNHG17. Further results showed that miR-328-3p expression was negatively correlated with SNHG17 expression in RCC tissues from our cohort and TCGA-KIRC. The binding function of SNHG17 and miR-328-3p was also confirmed by luciferase report assay and RIP assays. Recent reports have found that miR-328-3p functions as a tumor suppressive gene in several types of human malignancies, such as osteosarcoma, liver cancer, bladder cancer [24–27]. However, the expression levels and biological roles of miR-328-3p in RCC have not been reported. Our present data demonstrated the tumor-suppressive role of miR-328-3p in RCC cells *in vitro* and the downregulation of miR-328-3p in RCC tissue specimens and cell lines. Moreover, our rescue experiment found that miR-328-3p mediated the biological function of SNHG17 in RCC cells.

To examine the downstream targets of miR-328-3p in RCC cell lines, we used three independent online programs and TCGA database to screen putative targets. H2AX was identified to share the target gene of miR-328-3p. Furthermore, luciferase reporter assays revealed that miR-328-3p was able to recognize and directly target the wt 3′-UTR, but not the mut 3’UTR of H2AX. Overexpression of miR-328-3p repressed SNHG17 mRNA and protein expression, while miR-328-3p silencing elevated SNHG17 expression. In RCC tissue specimens, we found that H2AX inversely correlated with miR-328-3p but positively with SNHG17 expression. Notably, the further rescue experiments verified the regulatory axis of SNHG17/miR-328-3p/H2AX in the malignant biological phenotypes of RCC cell lines. H2AX isa variant of the core histone H2A. A phosphorylated form of H2AX, named gamma-H2AX, has become an early marker of DSBs formation, which signifies genomic instability, and regulates molecules needed for DNA repair [26, 28, 29]. We examined the effects of SNHG17 on gamma-H2AXexpression, and found that SNHG17 positively modulated gamma-H2AXexpression, indicating that SNHG17 might affect genomic stability. Similarly, Han et al. showed that SNHG17 mediated Helicobacter pylori infection-induced DSBs [20]. Moreover, H2AX had been proved to be involved in cancer initiation and progression [30, 31]. Also, H2AX plays a vital role in regulating tumor cell proliferation [32] and angiogenesis [33].

This study is the first to demonstrate the function and mechanism of SNHG17 in RCC progression. Our study reveals thatSNHG17 could function as a miRNA sponge to positively regulate H2AX expression via binding miR-328-3p and subsequently promotes the progression of RCC.

## Supplementary Materials

Supplementary Figures

Supplementary Tables
