# Supplementary material for: LncRNA SNHG17 promotes tumor progression and predicts poor survival in human renal cell carcinoma via sponging miR-328-3p
Source: Aging (Albany NY). 2021 Sep 8;13(17):21232–50. doi: 10.18632/aging.203440 (PMC8457601; doi:10.18632/aging.203440)
Supplement: Supplementary Figures [file aging-13-203440-s001.pdf]

SUPPLEMENTARY FIGURES

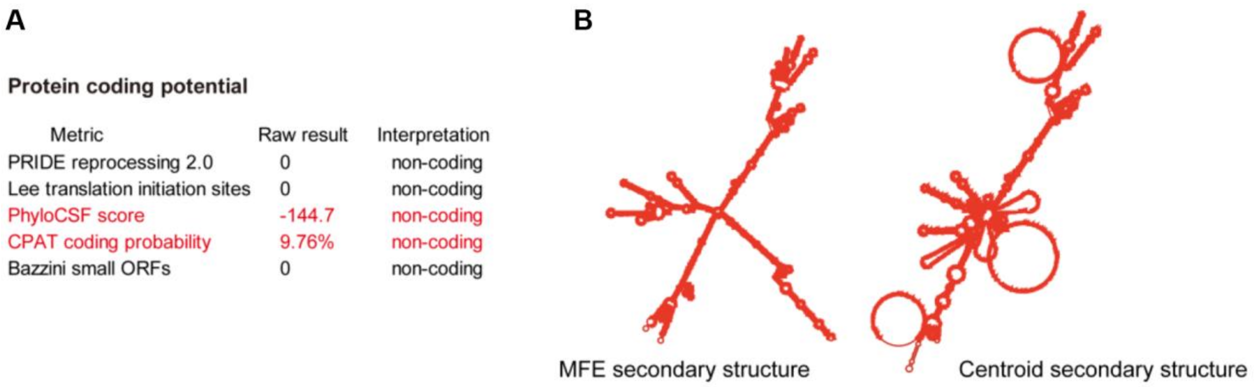

Supplementary Figure 1. (**A**) Protein coding potential of SNHG17 predicted using LNCipedia (https://lncipedia.org). (**B**) The RNA secondary structure of SNHG17 predicted using RNAfold Webserver (http://rna.tbi.univie.ac.at/) based on minimum free energy (MFE) and partition function.

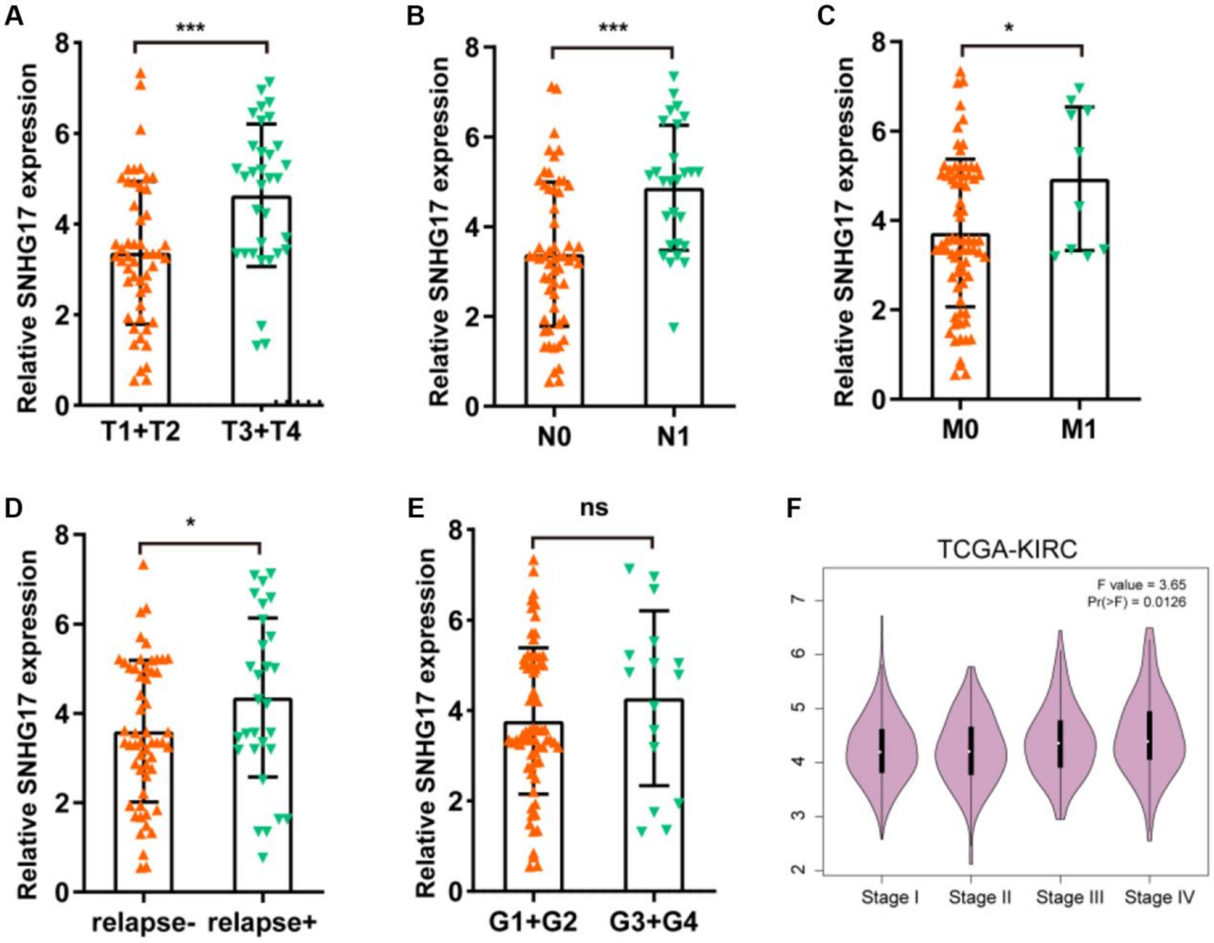

Supplementary Figure 2. Clinical significance of SNHG17 in RCC. (**A**–**E**) qRT-PCR assay analysis of the association between expression of SNHG17 and T stage (**A**) node invasion (**B**), metastasis status (**C**), relapse status (**D**), and tumor grade (**E**). Data were obtained using the $2^{-\Delta\Delta CT}$ method and were normalized to GAPDH levels. (**F**) Expression of SNHG17 was significantly associated with aggressive TNM stage according to TCGA-KIRC dataset. ns, not significant; $^{*}P < 0.05$; $^{**}P < 0.01$; $^{***}P < 0.001$.

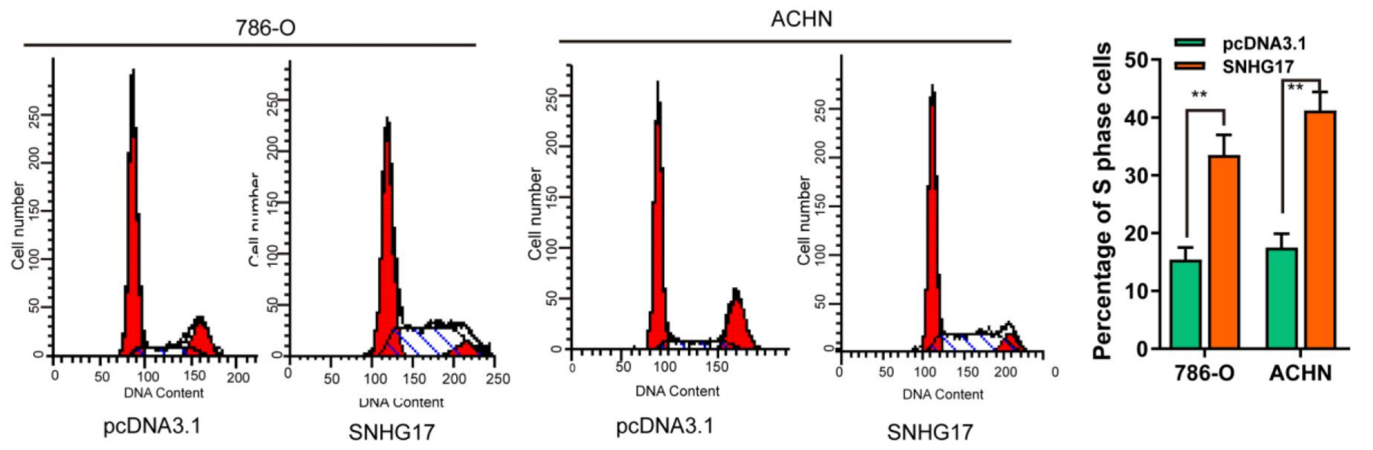

Supplementary Figure 3. Flow cytometry assays were used to detect cell cycle distribution following RCC cells with SNHG17 overexpression.

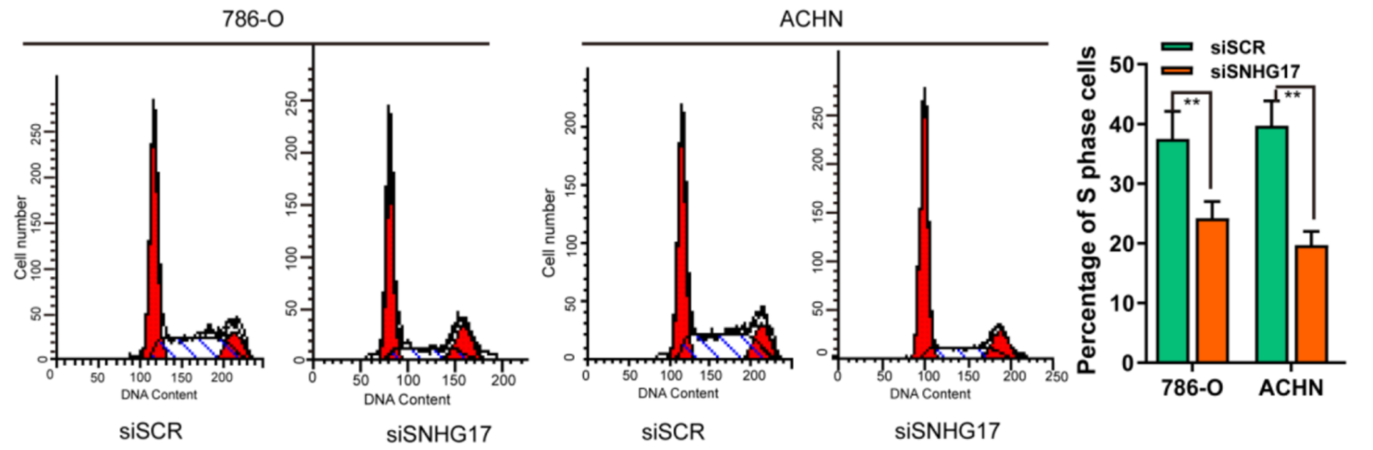

Supplementary Figure 4. Flow cytometry assays were used to detect cell cycle distribution following RCC cells with SNHG17 silencing.

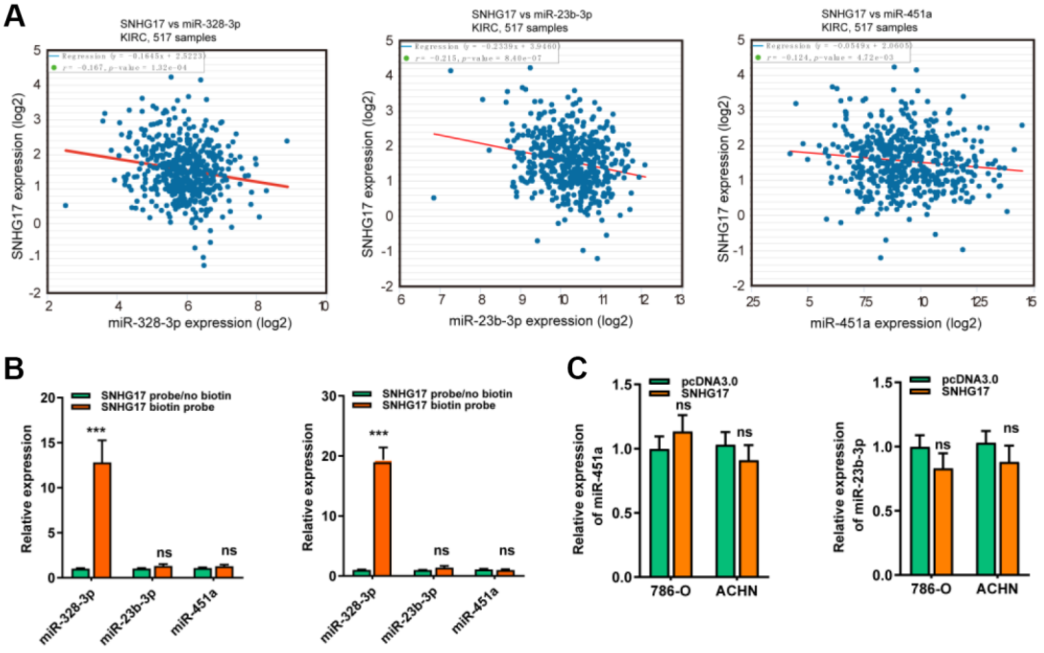

Supplementary Figure 5. (**A**) MiR-328-3p, miR-23b-3p, miR-451a were suggested to possess a negative correlation with SNHG17 in human RCC tissue samples according to TCGA-KIRC dataset. (**B**) RNA pull down assay showed that only miR-328-3p was significantly pulled down by biotinylated SNHG17 in RCC cell lines. (**C**) qRT-PCR assay analysis of the expression levels of miR-23b-3p or miR-451a in 786-O and ACHN cell lines after transfection with SNHG17 overexpression vectors. ns, not significant; $^{**}P < 0.01$; $^{***}P < 0.001$.

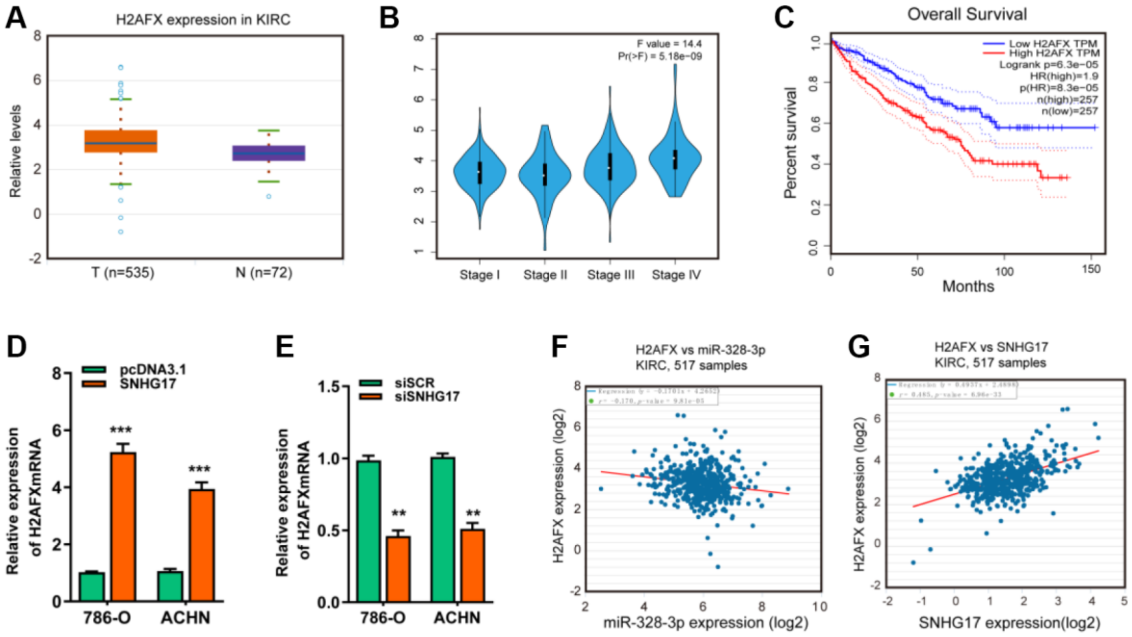

Supplementary Figure 6. (**A**–**C**) According to TCGA-KIRC dataset, H2AX expression was upregulated, associated with advanced TNM stage and poor survival. (**D**–**E**) qRT-PCR assay analysis of the expression levels of H2AX in RCC cells after transfection with the indicated vectors. (**F**–**G**) Spearman's correlation analysis of the association between H2AX expression and miR-328-3p (**F**) or SNHG17 (**G**) expression in RCC tissues from TCGA-KIRC dataset. ns, not significant; $^{**}P < 0.01$; $^{***}P < 0.001$.
