# Supplementary material for: LncRNA SNHG17 promotes tumor progression and predicts poor survival in human renal cell carcinoma via sponging miR-328-3p
Source: Aging (Albany NY). 2021 Sep 8;13(17):21232–50. doi: 10.18632/aging.203440 (PMC8457601; doi:10.18632/aging.203440)
Supplement: Supplementary Tables [file aging-13-203440-s002.pdf]

SUPPLEMENTARY TABLES

Supplementary Table 1. Clinicopathologic characteristics of the renal cell carcinoma patients.

Parameters		%
Age, years		
<55	37	43.4
≥55	47	56.6
Gender		
Male	53	69.7
Female	31	30.3
Pathological T stage		
pT1 + pT2	51	60.7
pT3 + pT4	33	39.3
Pathological N stage		
pN0	57	67.9
pN1	27	32.1
Distant metastasis		
No	74	88.1
Yes	10	11.9
TNM Stage		
I	8	9.5
II	38	45.2
III	28	33.3
IV	10	11.9
Grade		
1 + 2	67	79.8
3 + 4	16	19.0

Supplementary Table 2. Primers for qRT-PCR.

	Forward premier (5′–3′)	Reverse premier (5′–3′)
H2AX	CGGCAATGCTGGAGTACCTCA	AGCTCCTCCTCGTTGCGGATG
GAPDH	GGTGAAGGTCGGAGTCAACGG	GAGGTCAATGAAGGGGTCATTG
U6	GCTTCGGCAGCACATATACT	GTGCAGGGTCCGAGGTATTC
SNHG17	AGAGAATGGAGAGTGAGGCTACC	CCAGGCATGGACAGAGGGAT
